# The SCF^Skp2^ ubiquitin ligase complex modulates TRAIL-R2-induced apoptosis by regulating FLIP(L)

**DOI:** 10.1038/s41418-020-0539-7

**Published:** 2020-04-20

**Authors:** Jamie Z. Roberts, Caitriona Holohan, Tamas Sessler, Jennifer Fox, Nyree Crawford, Joel S. Riley, Hajrah Khawaja, Joanna Majkut, Emma Evergren, Luke M. Humphreys, Jennifer Ferris, Catherine Higgins, Margarita Espona-Fiedler, Paul Moynagh, Simon S. McDade, Daniel B. Longley

**Affiliations:** 1 Centre for Cancer Research and Cell Biology, Queen’s University Belfast, Belfast, UK; 2 Department of Biology, National University of Ireland Maynooth, Kildare, Ireland; 3 Centre for Experimental Medicine, Queen’s University Belfast, Belfast, UK

**Keywords:** Cancer, Cancer

## Abstract

TRAIL-R2 (DR5) is a clinically-relevant therapeutic target and a key target for immune effector cells. Herein, we identify a novel interaction between TRAIL-R2 and the Skp1-Cullin-1-F-box (SCF) Cullin-Ring E3 Ubiquitin Ligase complex containing Skp2 (SCF^Skp2^). We find that SCF^Skp2^ can interact with both TRAIL-R2’s pre-ligand association complex (PLAC) and ligand-activated death-inducing signalling complex (DISC). Moreover, Cullin-1 interacts with TRAIL-R2 in its active NEDDylated form. Inhibiting Cullin-1’s DISC recruitment using the NEDDylation inhibitor MLN4924 (Pevonedistat) or siRNA increased apoptosis induction in response to TRAIL. This correlated with enhanced levels of the caspase-8 regulator FLIP at the TRAIL-R2 DISC, particularly the long splice form, FLIP(L). We subsequently found that FLIP(L) (but not FLIP(S), caspase-8, nor the other core DISC component FADD) interacts with Cullin-1 and Skp2. Importantly, this interaction is enhanced when FLIP(L) is in its DISC-associated, C-terminally truncated p43-form. Prevention of FLIP(L) processing to its p43-form stabilises the protein, suggesting that by enhancing its interaction with SCF^Skp2^, cleavage to the p43-form is a critical step in FLIP(L) turnover. In support of this, we found that silencing any of the components of the SCF^Skp2^ complex inhibits FLIP ubiquitination, while overexpressing Cullin-1/Skp2 enhances its ubiquitination in a NEDDylation-dependent manner. DISC recruitment of TRAF2, previously identified as an E3 ligase for caspase-8 at the DISC, was also enhanced when Cullin-1’s recruitment was inhibited, although its interaction with Cullin-1 was found to be mediated indirectly via FLIP(L). Notably, the interaction of p43-FLIP(L) with Cullin-1 disrupts its ability to interact with FADD, caspase-8 and TRAF2. Collectively, our results suggest that processing of FLIP(L) to p43-FLIP(L) at the TRAIL-R2 DISC enhances its interaction with co-localised SCF^Skp2^, leading to disruption of p43-FLIP(L)’s interactions with other DISC components and promoting its ubiquitination and degradation, thereby modulating TRAIL-R2-mediated apoptosis.

## Introduction

Apoptosis plays a key role in maintaining normal tissue homeostasis and preventing disease [1]. Apoptosis is orchestrated by a family of cysteine proteases, the caspases [2]. The complex formed following activation of the TRAIL-R1/R2 (DR4/DR5) death receptors is called the death-inducing signalling complex (DISC), consisting of the receptors, the adaptor molecule FADD, procaspase-8 and FLIP [3]. FADD recruits procaspase-8 into this complex [4]. Procaspase-8 dimerization results in conformational changes in its catalytic domains that are necessary for its activation [5]. Two main splice forms of FLIP have been identified: a long form (FLIP(L)) and a short form (FLIP(S)), both of which can be recruited to the DISC and related complexes (such as TNFR1 Complex II and the ripoptosome) where they form heterodimers with procaspase-8 and regulate its processing and activity [4]. Therefore, the relative amounts of FLIP(S) and FLIP(L) recruited to the DISC is a key determinant of cell fate.

Although immune effector cells express TRAIL, rather than being lost, TRAIL-R2 is commonly over-expressed on malignant cells [6–8]. In pre-clinical studies, TRAIL was demonstrated to activate apoptosis selectively in cancer cells compared to normal cells [9, 10]. Thus, TRAIL receptors are promising targets for selective targeting of tumor cells. Whilst some promising activity was seen in early clinical trials of 1st generation TRAIL-R-targeted therapeutics [11, 12], overall single-agent activity was limited. Emerging 2nd generation TRAIL-R2-selective agents are multivalent, exhibit potent pre-clinical activity and have now reached early phase clinical trials. It is therefore important to understand how apoptosis signalling from this receptor is regulated, not only to understand TRAIL-mediated anti-tumor immunity, but also to predict and enhance response to 2nd generation TRAIL-R2 agonists.

**Fig. 1:**
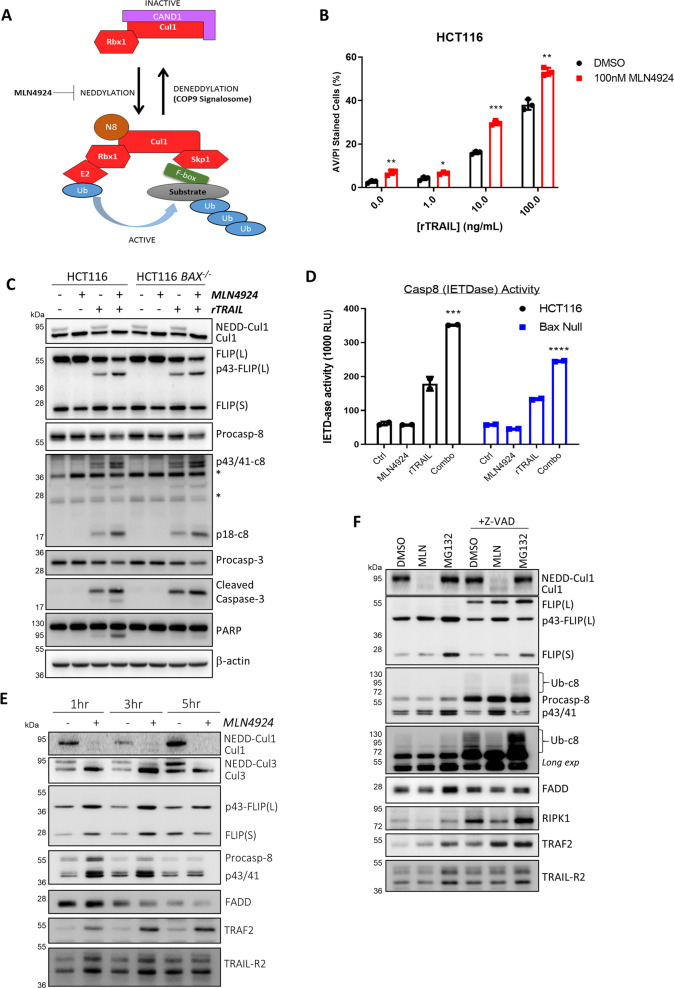
The NEDDylation inhibitor MLN4924 enhances TRAIL-induced apoptosis. **a** Schematic diagram of the Skp1-Cullin-1-F-box protein (SCF) Cullin-RING Ligase (CRL) complex and its regulation by NEDDylation. **b** High content microscopy analysis of cell death in HCT116 cells pre-treated with MLN4924 (100 nM) for 1 h, before treatment with rTRAIL for an additional 24 h. A student’s *T* test was performed between DMSO and MLN4924-treated samples (Error bars = SEM, ****p < 0.0001, ***p < 0.001, **p < 0.01, *p < 0.05, ns = *p* > 0.05). **c** Western blot analyses and matched caspase-8 activity assays (**d**) in HCT116 parental and Bax-deficient cells co-treated with MLN4924 (100 nM) and rTRAIL (10 ng/mL) for 6 h. A two-way ANOVA was performed between Ctrl vs rTRAIL and MLN4924 vs Combo in each cell line (Error bars = SEM, ****p < 0.0001, ***p < 0.001, **p < 0.01, *p < 0.05, ns = *p* > 0.05). **e** Western blot analyses of TRAIL-R2 DISC IP in HCT116 BAX-null cells pre-treated with MLN4924 (100 nM) for 1 h, prior to activation of the TRAIL-R2 receptor for the indicated times. **f** Western blot analysis of TRAIL-R2 DISC IP in HCT116 BAX-null cells treated for 1 h with MLN4924 (100 nM) or MG132 (10 µM) prior to activation of the TRAIL-R2 receptor for 1 h; cells were pre-treated with 20 µM zVAD-fmk pan-caspase inhibitor for 2 h as indicated.

NEDD8 is a 9 kDa ubiquitin-like protein (UBL) [13, 14]. Just like the ubiquitination cascade, the NEDDylation cascade relies on an E1/E2/E3 enzyme system; this results in the formation of an isopeptide bond between the C-terminus of NEDD8 (Gly76) and a substrate’s lysine residue. The most researched NEDDylation substrates are the Cullin-Ring E3 Ligase (CRL) family of E3 ubiquitin ligases [15, 16]. CRLs are multimeric ubiquitin E3 ligases made up of three components (Fig. 1a): the Cullin, which acts as a scaffold; the C-terminal RING finger domain-containing Rbx1/2 catalytic subunit; and the N-terminal substrate-binding domain, which consists of an adaptor (that binds the Cullin) and substrate receptor (that binds the target protein). The activity of CRLs is critically dependent on NEDDylation: when a CRL is NEDDylated, it is active. The CRLs account for roughly 20% of all proteasomal degradation within the cell [17, 18], and many CRL proteins, especially the F-box family of proteins, have been linked to tumorigenesis [19]. It has become increasingly apparent how crucial ubiquitination is for Death Receptor signalling [20], including the TRAIL-R2 DISC, at which caspase-8 activation has been shown to be regulated by Cullin-3 [21].

In this study, we identify the Skp1-Cullin-1-F-box protein containing Skp2 complex (SCF^Skp2^) as a TRAIL-R2-associated complex that regulates apoptosis signalling from the DISC by interacting with and modulating turnover of FLIP(L).

## Results

### The NEDDylation inhibitor MLN4924 enhances rather than inhibits TRAIL-induced apoptosis

Cullin-3 has been reported to interact with the DISC, where it promotes K63-linked ubiquitination of the p10-subunit of caspase-8, which then results in recruitment of active caspase-8 into ubiquitin-rich foci that in turn enhances its enzymatic activity and apoptosis induction [21]. As inhibition of NEDD8-activating E1 enzyme (NAE1) blocks Cullin NEDDylation thereby rendering CRLs inactive (Fig. 1a), it was surprising that MLN4924 (TAK4924/Pevonedistat), a selective small-molecule inhibitor of the NAE1 [22], significantly enhanced rather than inhibited TRAIL-induced apoptosis in HCT116 colorectal cancer (CRC) (Fig. 1b and Supplementary Fig. 1a), A549 non-small cell lung cancer (NSCLC) and HT29 CRC (Supplementary Fig. 1b) cell lines. Moreover, in HCT116 cells co-treated with TRAIL and MLN4924 for 6 h, enhanced levels of the active subunit of caspase-8 (p18) were detected by Western blot, and this correlated with enhanced PARP cleavage and caspase-8 activity (Fig. 1c, d). In addition, the levels of p43-FLIP(L) and p43/41-caspase-8 were elevated in MLN4924/TRAIL co-treated cells (Fig. 1c). Furthermore, the effects of MLN4924 on caspase-8 and FLIP(L) processing were not due to downstream apoptosis signalling as almost identical effects were observed in BAX-deficient cells (Fig. 1c, d), although full processing of caspase-3 leading to PARP cleavage in this model was inhibited (this was expected as HCT116 cells are “Type 2”, meaning they require involvement of the mitochondrial apoptotic pathway to amplify the apoptotic signal from the DISC [23]). These effects were also maintained after 24 h, including maintenance of NEDDylation inhibition as indicated by the absence of NEDDylated Cullin-1 (Supplementary Fig. 1c).

### Identification of Cullin-1 as a novel TRAIL-R2 DISC-interacting protein

Based on these results, we hypothesized that another NEDDylation-dependent Cullin may modulate TRAIL-induced apoptosis at the DISC. Using a DISC IP assay for stimulating TRAIL-R2 DISC formation and then assessing its components [24], we found that, in addition to Cullin-3, Cullin-1 was also present (Fig. 1e and Supplementary Fig. 1d). Moreover, Cullin-1 was primarily recruited in its NEDDylated form, as pre-treatment with MLN4924 almost completely abrogated its DISC recruitment. A very small amount of non-NEDDylated Cullin-1 was still detected at the DISC in MLN4924-treated cells at 1 and 3 h. By comparison, Cullin-3 was detected equally in its NEDDylated and non-NEDDylated forms at the DISC, and was still present (predominantly in its non-NEDDylated form) following MLN4924 pre treatment. Thus, it appears that de-NEDDylated Cullin-1 is not retained at the DISC to the same extent as de-NEDDylated Cullin-3. In addition, NEDDylated Cullin-3 was almost completely lost; however, a corresponding increase in non-NEDDylated Cullin-3 was observed, suggesting that the total amount of Cullin-3 at the DISC is largely unaffected by NEDDylation status, whereas this is crucial for Cullin-1 recruitment. The pre treatment with MLN4924 also affected the core DISC components FLIP(L) (detected as its caspase-8 processed form p43-FLIP(L)) and caspase-8 (detected as its pro-form and partially processed p41/43-forms), the levels of both of which were significantly enhanced at the 1 and 3 h timepoints (Fig. 1e). FLIP(S) recruitment was also somewhat increased at these timepoints. Although FADD levels decreased over time, its levels were similar in control and MLN4924-treated samples within each timepoint. The levels of another DISC component, TRAF2, which has been reported to be a negative regulator of caspase-8 activity at the TRAIL-R2 DISC [25], were markedly enhanced by MLN4924 treatment.

Proteasome inhibitors have been reported to enhance DISC-mediated caspase-8 activation by inhibiting TRAF2-mediated ubiquitination of its large catalytic subunit [25]. We, therefore, compared the impact of MLN4924 and MG132 on TRAIL-R2 DISC assembly (Fig. 1f and Supplementary Fig. 1e). Treatment with both MLN4924 and MG132 enhanced the levels of both FLIP(L) and TRAF2 detected at the TRAIL-R2 DISC. MG132 and to a lesser extent MLN4924 also enhanced FLIP(S) levels at the DISC. Another notable effect of MLN4924 was to reduce the levels of poly-ubiquitinated caspase-8 detectable at the DISC. This is consistent with inhibition of Cullin-3-mediated caspase-8 ubiquitination; however, if Cullin-3-mediated ubiquitination enhances caspase-8 activity as has been reported [25], we would have expected to see a decrease in TRAIL-induced caspase-8 activation and apoptosis in MLN4924-treated cells rather than the observed increases (Fig. 1b–d). Treatment with MLN4924 also decreased the levels of RIPK1 detected at the DISC, a major substrate of the DISC-bound FLIP(L):caspase-8 heterodimer. In the presence of the pan-caspase inhibitor zVAD-fmk, it was easier to visualise the effects of MLN4924, with enhanced levels of p43-FLIP(L) and TRAF2, decreased levels of poly-ubiquitinated caspase-8 and decreased levels of RIPK1 were, again, observed. Due to its ability to partially inhibit DISC-bound caspase-8, unprocessed FLIP(L) was detected in the presence of zVAD-fmk, and both MLN4924 and MG132 enhanced the levels of this form of FLIP(L) present in the complex.

### The Skp1-Cullin-1-F-box protein containing Skp2 complex (SCF^Skp2^) is associated with TRAIL-R2

**Fig. 2:**
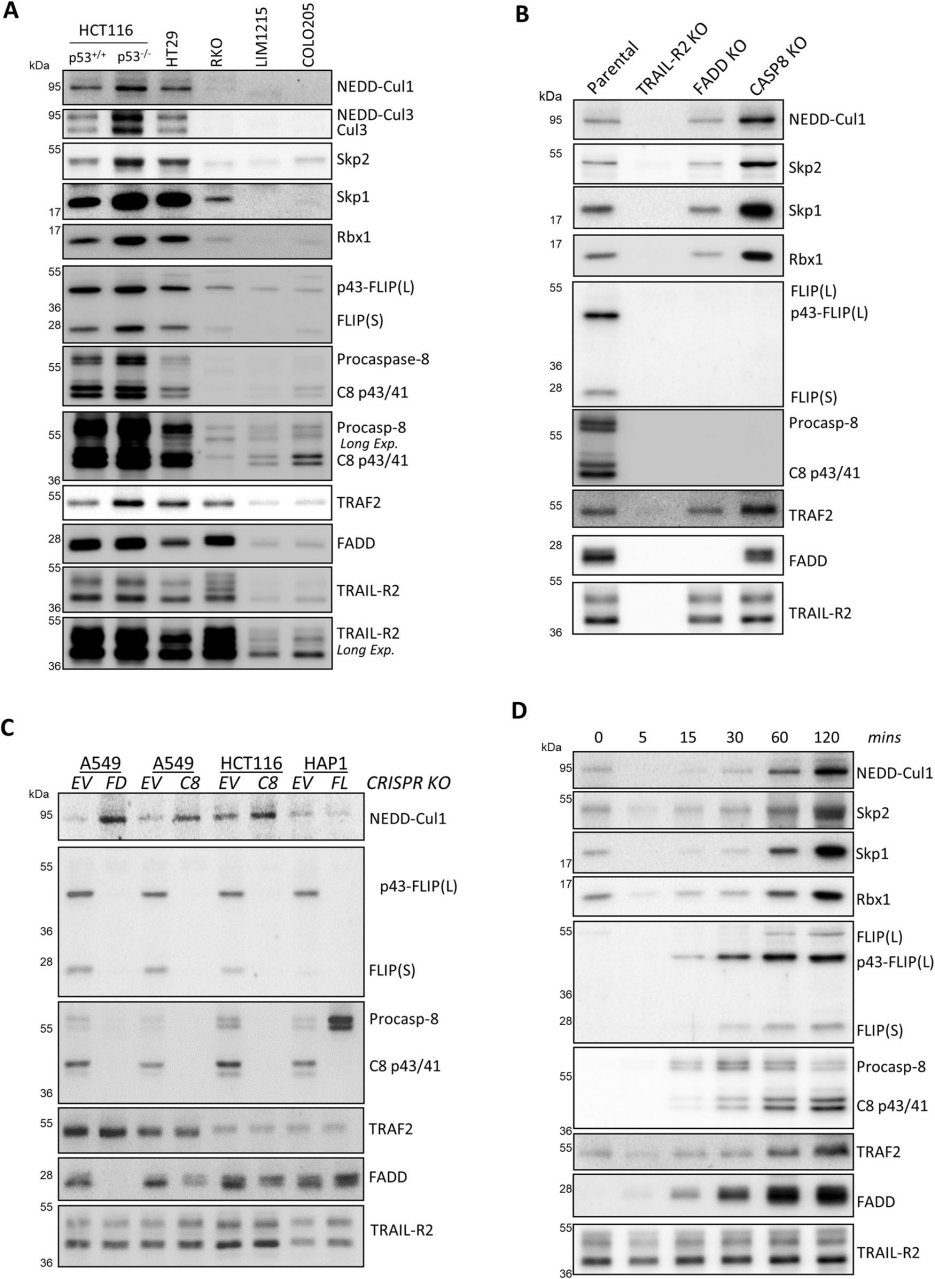
Identification of the SCF^Skp2^ complex as a novel TRAIL-R2 DISC-interacting protein. **a** Western blot analysis of TRAIL-R2 DISC IP (1 h) in a panel of colorectal cancer cell lines. **b** Western blot analysis of TRAIL-R2 DISC IP (1 h) in parental and TRAIL-R2/FADD/caspase-8 CRISPR knockout HCT116 cell lines. **c** Western blot analysis of TRAIL-R2 DISC IP (1 h) in parental and FADD/caspase-8 CRISPR knockout A549 cells; parental and caspase-8 CRISPR knockout HCT116 cells; parental and FLIP CRISPR knockout HAP1 cells. **d** Timecourse Western blot analysis of TRAIL-R2 DISC IP in HCT116 BAX-null cells.

To further investigate the role of Cullin-1 at the TRAIL-R2 DISC, we assessed its recruitment in a panel of colorectal cancer cell line models with differing degrees of TRAIL sensitivity. Cullin-1 was detected at the TRAIL-R2 DISC in TRAIL-sensitive HCT116 models and TRAIL-resistant HT29 cells, but was barely detectable in RKO, LIM1215 and COLO205 DISC IPs (Fig. 2a). Moreover, Cullin-1’s core binding partners Rbx1 and Skp1 were also detected in the HCT116 and HT29 models. Previous studies have linked resistance to TRAIL-induced apoptosis to Skp2[26], an F-box protein that is a part of the substrate receptor for the SCF CRL complex (Fig. 1a); therefore, we assessed whether Skp2 also associated with the TRAIL-R2 DISC. Skp2 was indeed detected, with higher levels at the TRAIL-R2 DISCs formed in the p53 mutant HT29 model and an isogenic p53 null HCT116 daughter cell line than in the p53 wild-type parental HCT116 model, an effect not attributable to higher levels of expression of the complex’s components (Supplementary Fig. 2a), suggesting a role for p53 in complex formation. Very low, if any, SCF^Skp2^ complex components could be detected in the DISCs formed by the LIM1215 and COLO205 cells (Fig. 2a), although these TRAIL-resistant models expressed low levels of the SCF^Skp2^ complex (Supplementary Fig. 2a) and cell surface TRAIL-R2 (as indicated by the amount of TRAIL-R2 immunoprecipitated). Although the TRAIL-resistant RKO model expressed all the SCF^Skp2^ components to a similar level as HCT116 and HT29 cell lines (Supplementary Fig. 2a), very little SCF^Skp2^ was detected at the DISC despite significant cell surface TRAIL-R2 expression (Fig. 2a). The levels of caspase-8 and FLIP at the DISC were also low at the RKO TRAIL-R2 DISC despite similar levels of expression (Supplementary Fig. 2a) and similar levels of FADD recruitment (Fig. 2a) to the other models. Recruitment of Cullin-3 followed a similar pattern to recruitment of Cullin-1.

Importantly, despite similar expression of all SCF^Skp2^ components (Supplementary Fig. 2b), none of the components of the SCF^Skp2^ complex were detected in HCT116 cells in which TRAIL-R2 had been deleted (Fig. 2b), confirming the specificity of the interaction. However, in cells lacking FADD, in which neither caspase-8 nor FLIP were recruited, Cullin-1 and the rest of the complex were detected at the DISC (Fig. 2b). Moreover, in HCT116 cells lacking caspase-8, recruitment of the SCF^Skp2^ complex was actually enhanced; in these cells, FADD was recruited to the DISC, but FLIP recruitment was impaired, consistent with observations made by us and others [27, 28]. In the A549 NSCLC model, lack of caspase-8 and FADD both enhanced Cullin-1 DISC recruitment (Fig. 2c and Supplementary Fig. 2c). FLIP (*CFLAR*) deletion in the HAP1 CML-derived cell line partially reduced Cullin-1 association with the TRAIL-R2 DISC as well as markedly inhibiting processing of procaspase-8 to its p41/43-forms.

In timecourse studies, components of the SCF^Skp2^ complex (and TRAF2) were all found to be associated with the unstimulated TRAIL-R2 (Fig. 2d; 0 min). Moreover, stimulation of TRAIL-R2 caused an initial loss of the receptor’s association with SCF^Skp2^, followed by re-association with similar dynamics as TRAF2 and canonical DISC proteins, FLIP, FADD and caspase-8 (Fig. 2d and Supplementary Fig. 2d). Collectively, these results indicate that, like TRAF2, the SCF^Skp2^ complex can associate with both the TRAIL-R2 pre-ligand-association complex (PLAC) and the ligand-activated TRAIL-R2 DISC. Furthermore, NEDDylated Cullin-1 was detected in membrane fractions, which were also enriched in TRAIL-R2 and canonical DISC and SCF^Skp2^ components (Supplementary Fig. 3a), indicating that these proteins can co-locate. Collectively, these results identify the SCF^Skp2^ complex as a novel TRAIL-R2-associated complex.

### Cullin-1 can regulate the composition and signalling outcomes of the TRAIL-R2 DISC

**Fig. 3:**
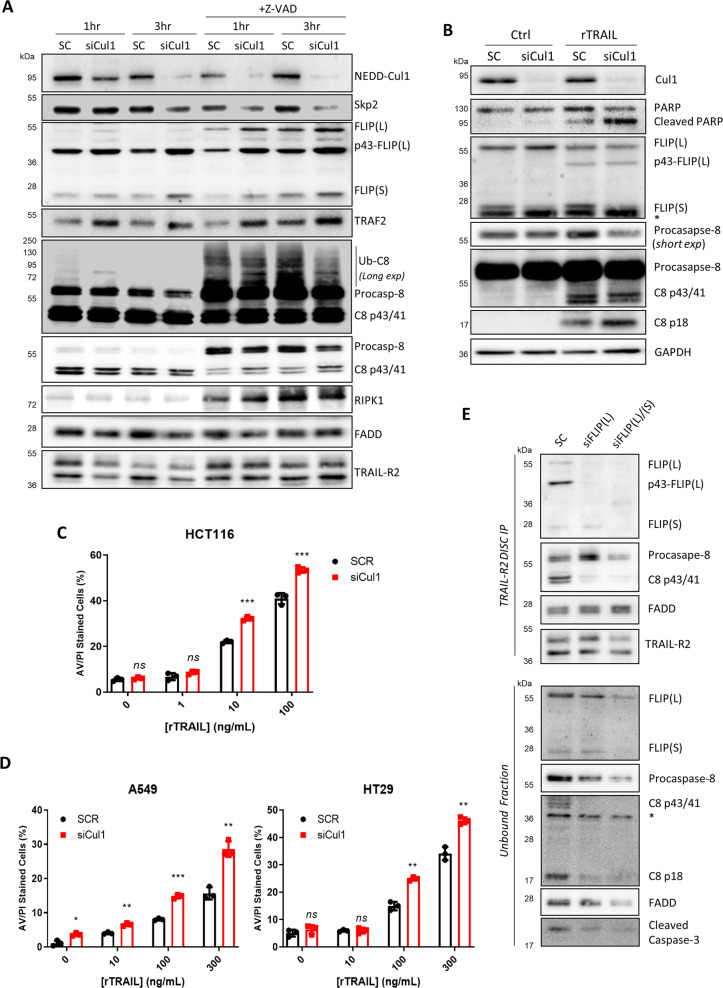
Cullin-1 inhibits TRAIL-induced apoptosis in HCT116 cells. **a** Western blot analysis of TRAIL-R2 DISC IP in HCT116 BAX-null cells transfected for 48 h with 30 nM control (SC) or a pool of Cullin-1 targeted (siCul1) siRNAs prior to treatment with zVAD-fmk (20 µM) for 2 h and then activation of the TRAIL-R2 receptor for 1 and 3 h. **b** Western blot analyses of apoptosis proteins in HCT116 cells transfected with 10 nM control (SC) or Cullin-1-targeted (siCul1) siRNAs for 48 h prior to treatment with rTRAIL (10 ng/mL) for 6 h. High content microscopy analysis of cell death in (**c**) HCT116 cells and (**d**) A549 and HT29 cells transfected with 10 nM control (SC) or Cullin-1-targeted (siCul1) siRNAs for 24 h prior to treatment with rTRAIL for an additional 24 h. A student’s *T* test was performed between SC and siCul1 samples (Error bars = SEM, ****p < 0.0001, ***p < 0.001, **p < 0.01, *p < 0.05, ns = *p* > 0.05). **e** Western blot analysis of TRAIL-R2 DISC IP in HCT116 BAX-null cells transfected with 10 nM of a pool of FLIP(L)-specific or a pool of pan-FLIP siRNAs and treated with 10 µM zVAD-fmk for 16 h prior to DISC stimulation for 1 h.

As MLN4924 affects multiple substrates, not least Cullin-3, we assessed to what extent its effects on TRAIL-R2 composition and signalling are Cullin-1-dependent using a Cullin-1-directed siRNA pool (siCul1). At the DISC, increases in p43-FLIP(L) and TRAF2 were observed in siCul1-transfected cells (Fig. 3a and Supplementary Fig. 3b). Similar effects were observed in the presence of zVAD-fmk along with increases in (the now observable) unprocessed FLIP(L) and a slight increase in FLIP(S). siCul1 had less impact on poly-ubiquitinated caspase-8 than MLN4924 (Fig. 3a), consistent with this effect of the NEDDylation inhibitor being mediated via Cullin-3 as previously reported [25], and its impact on RIPK1 levels was small. Additionally, depleting had little effect on Cullin-3 recruitment to the DISC (Supplementary Fig. 3c); this suggests that Cullin-1 and Cullin-3 DISC recruitment are not inter-dependent. Collectively, these results indicate that the interaction of Cullin-1 with the TRAIL-R2 DISC mainly affects the levels of FLIP(L) and TRAF2 associated with the complex.

The impact of these changes on TRAIL-induced apoptosis was similar to those observed for MLN4924, with increased levels of cleaved PARP and p18-caspase-8 (Fig. 3b) and significantly enhanced cell death induction (Fig. 3c). Similar results were obtained using Skp2-directed siRNAs (siSkp2) (Supplementary Fig. 3d) and in other models (A549 and HT29) in which Cullin-1 was detected at the DISC (Fig. 3d).

Analogous to the effects of CRISPR-mediated deletion of FLIP in HAP1 cells (Fig. 2c), siRNA-mediated down-regulation of FLIP(L) in HCT116 cells inhibited procasapse-8 processing at the TRAIL-R2 DISC thereby reducing the levels of fully processed p18-caspase-8 detected in the unbound fraction (Fig. 3e). This indicates that FLIP(L) facilitates procaspase-8 processing in this setting and is consistent with the impact of MLN4924 treatment and Cullin-1 silencing, both of which enhance FLIP(L) and p43-FLIP(L) levels at the TRAIL-R2 DISC and promote TRAIL-induced apoptosis. Furthermore, although Cullin-1 depletion enhanced MLN4924-induced apoptosis, the extent of sensitization to TRAIL-induced apoptosis was of a similar magnitude to that induced by MLN4924 alone and siCul1 alone (Supplementary Fig. 3e), consistent with the primary impact of MLN4924 on TRAIL sensitivity being mediated by its inhibitory effects on Cullin-1.

### The SCF^Skp2^ complex interacts with FLIP(L)

**Fig. 4:**
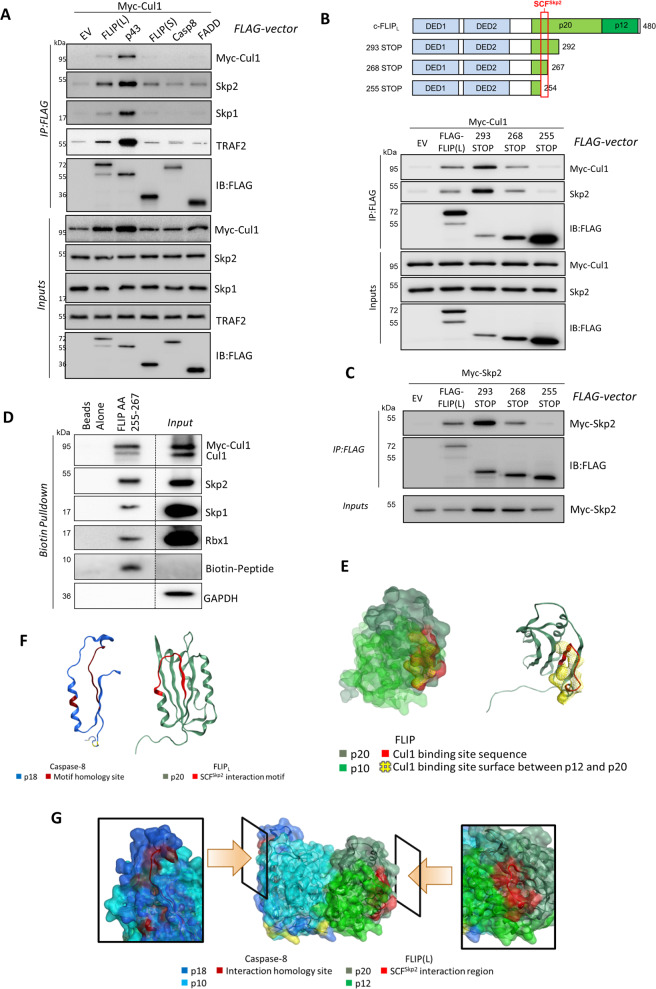
Mapping the SCF^Skp2^ interaction site within FLIP(L). **a** Co-IP analysis of the interaction of Cullin-1 with full-length FLIP(L), p43-FLIP(L) (p43), FLIP(S), procaspase-8 (Casp8) and FADD; interactions with endogenous Skp1, Skp2 and TRAF2 were also assessed. **b** Schematic diagram of FLIP expression constructs; the death effector domains (DEDs) and large (p20) and small (p12) subunits of the pseudo-caspase domain are highlighted. The red box indicates the region important for SCF^Skp2^ interaction, as deduced from Co-IP experiment mapping the interaction site between FLIP(L) and Cullin-1 and (**c**) Skp2. **d** Biotinylated peptide of FLIP(L)’s SCF^Skp2^ interaction site pull-down experiment demonstrating the interaction of this region with the SCF^Skp2^ complex. **e** Space-filling and ribbon models of the Cullin-1 interaction site in the large p20-subunit of FLIP(L) and its spatial orientation with respect to the p12 small subunit. **f** Ribbon models of the Cullin-1 interaction site in FLIP(L) and the corresponding region of caspase-8. **g** Model derived from the crystal structure (*PDB ID: 3H11*) of the large and small catalytic domains of the caspase-8/FLIP(L) heterodimer. The position of the SCF^Skp2^ complex interaction site and the equivalent (non-contiguous) residues in caspase-8 are highlighted in red. A frontal view of the heterodimer interface is depicted in the middle; the “windows” show 90° rotations of the crystal structure, highlighting the SCF^Skp2^ interaction of FLIP(L) and its equivalent region in Procaspase-8.

We next assessed whether any of the core DISC components interact with components of the SCF^Skp2^ complex. In Co-IP experiments, we found that Cullin-1 interacts with FLIP(L) and TRAF2, but not FLIP(S), caspase-8 or FADD (Fig. 4a and Supplementary Fig. 4a). Endogenous Skp1 and Skp2 were also detected in these pull-downs; moreover, exogenous Skp2 was pulled down with FLIP(L), but not FLIP(S) (Supplementary Fig. 4b). Mapping studies localised the site of interaction between FLIP(L) and Cullin-1/Skp2 to a region between amino acids 211 and 350, which corresponds primarily to the large p20-subunit of its pseudo-caspase domain (Supplementary Fig. 4c–e). This is consistent with lack of interaction with FLIP(S), which does not contain this domain. Further mapping narrowed the interaction site to a region between amino acids 255 and 292 (Supplementary Fig. 5a–c). In these experiments, it was noticeable that the interaction between FLIP(L) and Cullin-1/Skp2 was significantly enhanced by deletion of the C-terminal region of FLIP(L), which contains the p12-subunit that is cleaved in a caspase-8-dependent manner at the DISC. Indeed, p43-FLIP(L)’s (p43) interaction with SCF^Skp2^ was significantly stronger than that of unprocessed FLIP(L) (Fig. 4a and Supplementary Fig. 4d, e). These results suggested that cleavage of FLIP(L) to its p43-form promotes its interaction with the SCF^Skp2^ complex.

We were subsequently able to fine map the SCF^Skp2^ interaction domain to amino acids 255-267 in FLIP(L) (Fig. 4b, c). Moreover, a Biotin-tagged peptide corresponding to this 13 amino acid region (plus additional flanking residues) interacted with all SCF^Skp2^ components in pull-down studies (Fig. 4d). Using a model of the large and small catalytic subunits of the caspase-8/FLIP(L) heterodimer derived from its crystal structure [29], we found that much of the 13 amino acid SCF^Skp2^ interaction region (red) in the p20-subunit of FLIP(L) is present on the protein surface and therefore available for forming protein–protein interactions, although some amino acids are buried in the interface with the p12-subunit (Fig. 4e–g). FLIP(L) is cleaved between its large (p20) and small (p12) catalytic subunits by caspase-8 to generate p43-FLIP(L) when it forms a heterodimer with caspase-8 at the DISC and related complexes; cleavage between these domains by caspase-8 at the DISC may enhance the surface exposure of the SCF^Skp2^ binding domain explaining the observed enhanced interaction between SCF^Skp2^ and p43-FLIP(L). This 13 amino acid region in FLIP(L) is not conserved in procaspase-8, which has a 21 amino acid insertion in this region (Fig. 4f, g and Supplementary Fig. 5d), consistent with the lack of interaction between SCF^Skp2^ and caspase-8 (Fig. 4a).

### FLIP(L) cleavage promotes its degradation

**Fig. 5:**
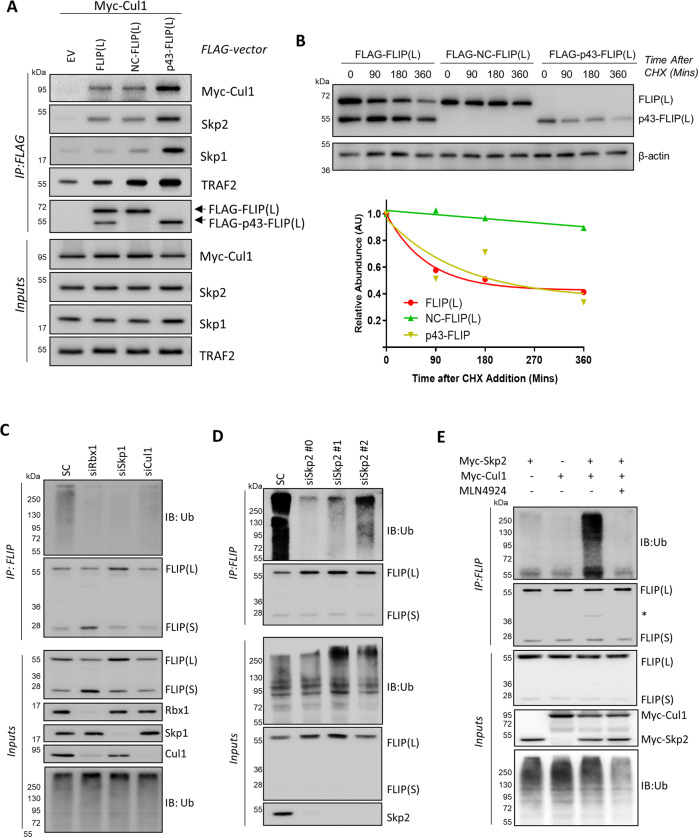
FLIP(L) cleavage promotes its interaction with SCF^Skp2^. **a** Co-IP analysis of the interaction of Cullin-1 with wild-type, non-cleavable (NC)-FLIP(L) and p43-FLIP(L). Interactions with endogenous Skp1, Skp2 and TRAF2 were also assessed. **b** Western blot assessment of degradation rates of wild-type-, NC- and p43-FLIP(L) using 100ug/mL cycloheximide (CHX). **c** Western blot of FLIP ubiquitination in HCT116 cells transfected for 48 h with siRNAs (20 nM) targeting Rbx1, Skp1 or Cullin-1. **d** Western blot analysis of FLIP ubiquitination in HCT116 cells transfected for 48 h with siRNAs (10 nM) targeting Skp2. **g** Impact of Cullin-1 and Skp2 over-expression on FLIP ubiquitination in the presence/absence of MLN4924 (300 nM, 3 h).

Given the proximity of FLIP(L)’s SCF^Skp2^ interaction site to its p12-domain, we further investigated the effect of FLIP(L)’s cleavage for its interaction with SCF^Skp2^ by generating a non-cleavable form of FLIP(L) (NC-FLIP(L), D376A). Again, p43-FLIP(L) interacted more strongly with components of the SCF^Skp2^ complex than the wild-type protein, whereas the NC-FLIP(L) interacted to the same extent as wild-type (Fig. 5a). In addition, NC-FLIP(L) was significantly more stable than FLIP(L) and p43-FLIP(L) (Fig. 5b). These results indicate that not only does cleavage of FLIP(L) to its p43-form promote its interaction with SCF^Skp2^, it also promotes its turnover, suggesting a role for SCF^Skp2^ in regulating FLIP(L) ubiquitination and degradation via the ubiquitin-proteasome system (UPS). In support of this, we found that RNAi-mediated down-regulation of Cullin-1, Skp1 and Rbx1 decreased ubiquitination of endogenous FLIP (Fig. 5c) as did Skp2 down-regulation (Fig. 5d). Furthermore, simultaneous over-expression of Cullin-1 and Skp2 enhanced ubiquitination of endogenous FLIP, which was reversed with MLN4924 treatment (Fig. 5e). Collectively, these results indicate a key role for the SCF^Skp2^ complex in regulating ubiquitination and turnover of FLIP(L).

### Cullin-1 competes with TRAF2, caspase-8 and FADD for binding to p43-FLIP(L)

**Fig. 6:**
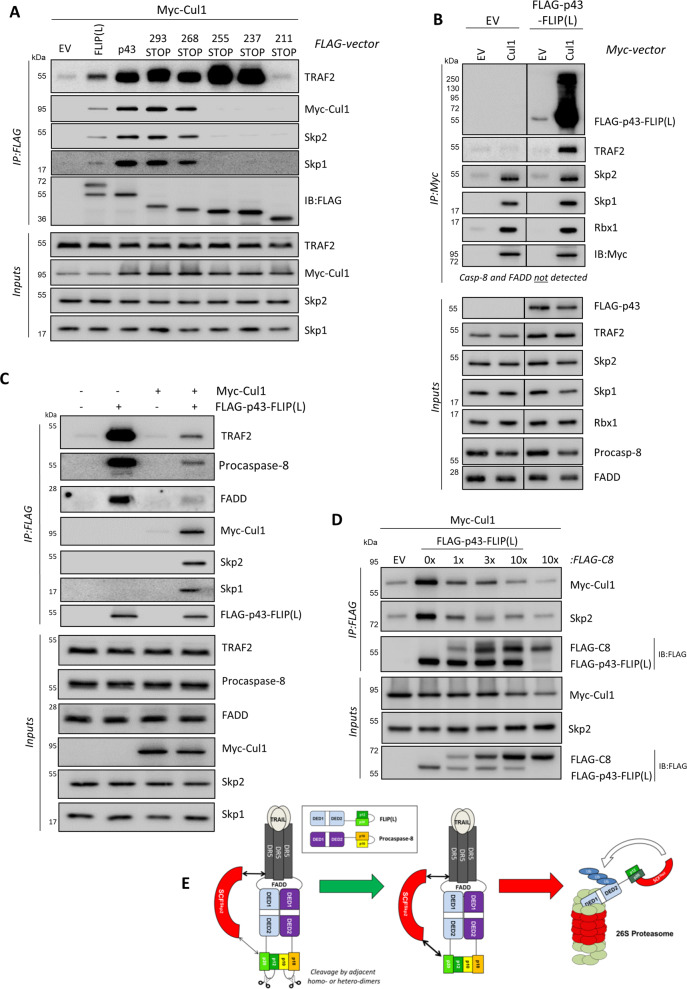
Cullin-1 competes with TRAF2, caspase-8 and FADD for binding to p43-FLIP(L). **a** Co-IP analysis of the interaction of Cullin-1 with full-length and truncated forms of FLIP(L); interactions with endogenous Skp1, Skp2 and TRAF2 were also assessed. **b** Co-IP analysis of the interaction of p43-FLIP(L) with Cullin-1 and endogenous Rbx1, Skp1 and Skp2. Components of the DISC were also assessed; neither caspase-8 nor FADD were detected, however TRAF2 was detectable, but only in cells co-transfected with p43-FLIP(L). **c** Co-IP analysis of the interaction of p43-FLIP(L) with endogenous FADD, caspase-8 and TRAF2 in the presence and absence of over-expressed Cullin-1; endogenous Skp1 and Skp2 were also assessed. **d** Co-IP analysis of the interaction of Myc-tagged Cullin-1 and endogenous Skp2 with FLAG-tagged p43-FLIP(L) in cells co-transfected with increasing amounts of FLAG-tagged procaspase-8 (C8). **e** *Schematic summary*: cleavage of FLIP(L) to p43-FLIP(L) at the TRAIL-R2 DISC (by proximal FLIP(L)/caspase-8 heterodimers or caspase-8 homodimers) enhances its interaction with Cullin-1, which disrupts its interaction with caspase-8, FADD and TRAF2 and promotes its ubiquitination and proteasomal degradation by SCF^Skp2^. This is facilitated by the constitutive interaction of SCF^Skp2^ with TRAIL-R2 and reduces the levels of the FLIP(L):caspase-8 heterodimeric enzyme at the DISC, thereby modulating TRAIL-induced caspase-8 activation and apoptosis induction.

We further investigated the interplay between TRAF2, FLIP(L) and components of the SCF^Skp2^ complex. Mapping studies re-confirmed the location of the SCF^Skp2^interaction site within FLIP(L) and demonstrated that the TRAF2’s interaction site is located in an adjacent region, between amino acids 211 and 236 of FLIP(L) (Fig. 6a and Supplementary Fig. 5e). TRAF2 co-immunoprecipitated with SCF^Skp2^ in the presence of p43-FLIP(L) or FLIP(L) but not FLIP(S), which does not contain this region, nor does caspase-8 or FADD (Fig. 4a), even though TRAF2 has been reported to act as an E3 ligase for caspase-8 at the DISC [25]. In mapping the TRAF2 binding site in FLIP(L), it was notable that the interaction was stronger once the Cullin-1 binding region was deleted (Fig. 6a). We subsequently found that TRAF2’s interaction with Cullin-1 is indirect and mediated via FLIP(L), as TRAF2 was only pulled down with Cullin-1 in the presence of p43-FLIP(L) (Fig. 6b). It is also worth noting that the fraction of p43-FLIP(L) associated with Cullin-1 (and Skp1, Skp2 and Rbx1) was extensively modified, potentially by polyubiquitin chains (Fig. 6b). Moreover, neither caspase-8 nor FADD were detected in this experiment, further confirming that these proteins do not interact with Cullin-1. In co-transfection experiments, the interaction of p43-FLIP(L) with TRAF2, FADD and procaspase-8 was significantly reduced in cells co-transfected with Cullin-1 (Fig. 6c) and complementary co-transfection experiments showed that procaspase-8 disrupted the interaction of p43-FLIP(L) with Cullin-1 (Fig. 6d and Supplementary Fig. 5f). These results are indicative of competition between canonical DISC proteins and SCF^Skp2^ for binding to p43-FLIP(L). Given the role of FLIP(L) in promoting procaspase-8 processing at the DISC (Fig. 3e), this competition between Cullin-1 and caspase-8, FADD and TRAF2 for binding to p43-FLIP(L) likely explains the observed Cullin-1-mediated inhibition of TRAIL-induced apoptosis (Fig. 3b–d) and the ability of MLN4924 to promote TRAIL-R2-mediated apoptosis (Fig. 1b–d and Supplementary Fig. 1a, b).

## Discussion

CRLs account for ~20% of all proteasomal degradation within the cell [17, 18], and many CRL proteins have been linked to tumourgenesis [19]. Cullin-3 interacts with the TRAIL-R2 DISC (which we confirmed) and has been reported to promote K63-linked ubiquitination of the p10-subunit of caspase-8, which enhances its enzymatic activity and apoptosis induction [21]. NEDDylation is required for CRLs to be active; however, using the NEDDylation inhibitor MLN4924, we found that TRAIL-induced apoptosis was enhanced rather than suppressed in cancer cells co-treated with MLN4924 and TRAIL, suggesting an alternative NEDDylation-dependent modulator of TRAIL-R2 apoptotic signalling. Indeed, we found that Cullin-1 and all the components of the SCF^Skp2^ complex associated with TRAIL-R2, both in its unstimulated (PLAC) and ligand-stimulated (DISC) forms.

Cullin-1 was predominantly detected in its NEDDylated (active) form at the TRAIL-R2 DISC, and inhibition or depletion of Cullin-1 increased p43-FLIP(L) levels at the DISC. By forming an enzymatically active heterodimer with p43/41-caspase-8, p43-FLIP(L) can inhibit apoptosis when present at high levels at the DISC or promote apoptosis when present at low levels [30]. In HCT116 cells, down-regulation of FLIP(L) inhibited procaspase-8 processing at the TRAIL-R2 DISC indicative of a pro-apoptotic role for FLIP(L) at the DISC in these cells under these conditions. Consistent with this, inhibition or down-regulation of Cullin-1 enhanced TRAIL-induced apoptosis in HCT116 cells and in A549 and HT29 cells, in which Cullin-1 was also detected at the TRAIL-R2 DISC; this suggests that by stabilising p43-FLIP(L) at the DISC, loss of Cullin-1 promotes p43-FLIP(L)-mediated processing of procaspase-8 and apoptosis induction.

FLIP is frequently over-expressed in cancer and is an important mediator of chemo- and radio-resistance as well as apoptosis induced by immune effector cells and therapeutic agonists [31–34]. The turnover of FLIP is regulated by the UPS, with half-lives of ~45 min for FLIP(S) and ~3 h for FLIP(L), indicative of distinct regulatory mechanisms for each [35]. A cytoplasmic complex termed the “FADDosome” formed in response to the antimetabolite 5-Fluorouracil was recently described containing FLIP, p53, ATR, caspase-10 and TRAF2, in which TRAF2 ubiquitinates FLIP(L) causing its degradation and activation of caspase-8-dependent apoptosis [36]. ER stress leading to JNK-mediated activation of ITCH has been reported to lead to ubiquitination and degradation of FLIP(L) [37, 38]. Having identified SCF^Skp2^ at the DISC, we explored its interaction with core DISC components and identified a specific interaction with FLIP(L), but not FLIP(S), caspase-8 or FADD. Moreover, down-regulation of SCF^Skp2^ components with siRNA decreased FLIP(L) ubiquitination, while over-expression of Cullin-1 and Skp2 increased FLIP(L) ubiquitination in a NEDDylation-dependent manner, providing the first evidence that FLIP(L) is regulated by the SCF^Skp2^ complex.

Mapping experiments revealed that SCF^Skp2^ interacted with amino acids 255-267 within the p20 pseudo-catalytic domain of FLIP(L), a region not present in FLIP(S), nor caspase-8. Importantly, a stronger interaction with SCF^Skp2^ was detected with p43-FLIP(L), which is generated by caspase-8 in FADD-dependent complexes such as the DISC. Molecular modelling of the FLIP(L):procaspase-8 heterodimer indicated that the Cullin-1 binding site in FLIP(L)’s p20-subunit is partly buried in the interface with its p12-subunit, suggesting that cleavage between these domains by caspase-8 at the DISC may enhance the surface exposure of the SCF^Skp2^ binding domain. Thus, our data suggest a model in which processing of FLIP(L) to p43-FLIP(L) at the TRAIL-R2 DISC enhances its interaction with co-localised SCF^Skp2^, leading to targeting of p43-FLIP(L) to the proteasome and reduced levels of the FLIP(L):caspase-8 heterodimer (Fig. 6e). This is consistent with the longer half-life of non-cleavable FLIP(L) and Cullin-1’s ability to disrupt p43-FLIP(L)’s interactions with FADD/caspase-8, indicative of competition between the SCF^Skp2^ complex and canonical DISC proteins for p43-FLIP(L) binding. Thus, in the absence of Cullin-1, enhanced levels of the p43-FLIP(L):caspase-8 heterodimeric enzyme at the TRAIL-R2 DISC would promote processing of its local substrates, including proximal procaspase-8 homodimers, thereby facilitating full processing of procaspase-8 to its fully active heterotetrameric form, leading to the enhanced levels of apoptosis that we observed in Cullin-1-depleted/inhibited cells.

FLIP(L)’s TRAF2 binding site, which we mapped to the linker region between DED2 and its p20-subunit, is adjacent to the Cullin-1 binding site, and the binding of TRAF2 to p43-FLIP(L) is clearly weakened by Cullin-1, suggesting competition between the SCF^Skp2^ complex and TRAF2 for binding to DISC-processed FLIP(L). Thus, in cells treated with MLN4924 or siCullin-1, DISC recruitment of TRAF2 was enhanced along with FLIP(L). Our findings agree with those of others [39, 40] that TRAF2 forms part of a FADD- and caspase-8-independent TRAIL-R2 PLAC and suggest that like other E3 ligases, c-Cbl/Cbl-b and A20, SCF^Skp2^ can also associate with this complex. Moreover, our findings agree with earlier studies which reported that TRAF2 interacts with p43-FLIP(L) at the DISC, leading to the latter’s stabilisation [41].

In conclusion, we report that SCF^Skp2^ can interact with TRAIL-R2 and regulate the turnover of FLIP(L) and its interactions with core DISC components. Whereas Cullin-3 regulates the TRAIL-R2 DISC by modulating caspase-8, our results indicate that Cullin-1 regulates the DISC primarily by modulating FLIP(L). Thus, our findings add to our understanding of how TRAIL-R2 signalling and FLIP are controlled with implications for therapeutically modulating this pathway.

## Methods

### Cell culture

HCT116 cells were cultured in McCoy’s 5 A Modified Medium, supplemented with 10% foetal calf serum (FCS) and 2mM L-glutamine (all from Life Technologies). HEK293T cells were maintained in Dulbecco’s Modified Eagle Medium (DMEM), supplemented with 10% foetal calf serum (FCS) and 1 mM Sodium Pyruvate (all from Life Technologies). COLO205 cells were maintained in Roswell Park Memorial Institute (RPMI) 1640 Medium (Sigma Aldrich, Gillingham, Dorset, UK), supplemented with 10% FCS (Life Technologies). A549, RKO, HT29 and LIM1215 cells were maintained in Dulbecco’s Modified Eagle Medium (DMEM) supplemented with 10% FCS (all from Life Technologies). HAP1 cells were maintained in Iscove’s Modified Dulbecco’s Medium (IMDM), supplemented with 10% foetal calf serum (all from Life Technologies). Cells were maintained in a tissue culture incubator (Sanyo Europe, Herts, UK) at 5% CO_2_ and 37 °C. HCT116, HCT116 BAX null, HCT116 p53 null, HT29, RKO, LIM1215, COLO205, A549 and HEK293T cells were obtained from American Type Culture Collection (ATCC, Manassas, VA, USA). HAP1 cells were obtained from Horizon Discovery (Cambridge, UK). Immediately after purchase, early passage stocks of each cell line were frozen down. After thawing, cells were kept for a limited number of passages and were regularly screened for the presence of mycoplasma using the MycoAlert Mycoplasma Detection Kit (Lonza, Basel, Switzerland).

### Oligonucleotide transfections

A 3:1 ratio of FuGENE® HD Transfection Reagent (Promega) (µL) to plasmid DNA (µg) was used for transfections. In 100 µL of OptiMEM (Life Technologies), FuGENE then plasmid DNA was added and left to incubate for 15 min at room temperature. The mixture was then added to cells in culture and was left for 24–48 h. For siRNA transfections, siRNA and Lipofectamine® RNAiMAX (Invitrogen) were added separately to OptiMEM (Life Technologies) before being mixed together and left to incubate at room temperature for 15 min. Culture medium was then added to the mixture, to allow total cell coverage, and was used to replace the medium on cells, which was then left to incubate at 5% CO_2_, 37 °C for 4 h. Further culture medium was then added, to normal culturing levels, and then cells were left for the indicated times.

### Immunoblotting

Cell lysates were prepared or immunoprecipitation (IP) samples were eluted using Loading buffer, subsequently followed by heating at 95 °C for 5 min. SDS-PAGE was then implemented to separate proteins by molecular weight using the Mini-PROTEAN 3 electrophoresis module apparatus (Bio-rad). The gels were then subsequently transferred using the Pierce Power Blotter (Thermo Scientific), with reagents and instructions used from the Trans-Blot® Turbo™ RTA Mini Nitrocellulose Transfer Kit (Bio-rad), for 11 min, at a constant of 25 V. Membranes were then incubated with the appropriate Primary antibody overnight at 4 °C and then a minimum of 3 h at 4 °C with the appropriate Secondary antibody. Protein expression was analysed and detected using the Western Lightning® Plus-ECL, Enhanced Chemiluminescence Substrate (PerkinElmer) and G:BOX Chemi XX6 gel doc system (Syngene). A list of antibodies is provided as a Supplementary Table.

### Ubiquitination assay

Cells grown in a P90 plate, typically collected at over 80% confluency, were washed once in ice-cold PBS to stop dynamic ubiquitination events from occurring; all procedures were then conducted at 4 °C for the same reason. Cells were transferred to tubes and were pelleted by centrifugation at 2400 rpm for 5 min; supernatant was subsequently removed. Cells were then lysed with 250 µL of SDS-free RIPA buffer (50 mM Tris pH 7.4, 150 mM NaCl, 5 mM EDTA, 1% Triton X-100, dH_2_O), supplemented with an EDTA free protease inhibitor cocktail, for 20 min before centrifugation at 13,00 rpm for 5 min, followed by the supernatant being transferred to fresh tubes. 25 µL of 10% SDS was added (Final concentration 1%) and the sample was heated at 95 °C for 5 min; to facilitate denaturation of the samples. The samples were then diluted with 750 µL of SDS-free RIPA buffer, supplemented with an EDTA free protease inhibitor cocktail, to dilute the SDS and allow the immunoprecipitation (IP) antibody to function efficiently; samples were then incubated with 1 µL of either FLIP (H-202) antibody (Santa Cruz biotechnology) or FLAG (M2) antibody (Sigma) overnight at 4 °C while rotating. Thirty microliters of the appropriate washed Dynabeads™ M-280 (Invitrogen) were then added to the samples and left to incubate for 6 h at 4 °C while rotating. The beads were then washed 5 times in SDS-free RIPA buffer and eluted in Loading buffer before being heated at 95 °C for 5 min.

### Co-Immunoprecipitation (Co-IP)

Cells grown in a P90 plate, typically collected at over 80% confluency, were washed once in ice-cold PBS to stop disruption of protein:protein interactions; all procedures were then conducted at 4 °C for the same reason. One milliliter of SDS-free RIPA buffer (50 mM Tris pH 7.4, 150 mM NaCl, 5 mM EDTA, 1% Triton X-100, dH_2_O), supplemented with an EDTA free protease inhibitor cocktail, was added onto cells within the plate and was left shaking for 20 min. Cell lysates were then transferred to a tube, centrifuged at 13,000 rpm for 5 min and the supernatant was transferred to a fresh tube. Either 1 µL of FLIP (H-202) antibody (Santa Cruz biotechnology)/Myc-Tag (9B11) antibody (Cell Signalling Technology) or 20 µL of FLAG (M2) Magnetic Beads (Sigma) were added to the cell lysates and were left to rotate overnight at 4 °C. The following day 15 µL of the appropriate washed Dynabeads™ M-280 (Invitrogen) were added to the samples and were left to rotate for a further 6 h. The beads were then washed 5 times with SDS-Free RIPA buffer and were eluted in Loading buffer, followed by heating at 95 °C for 5 min. However, if the FLAG (M2) Magnetic Beads were used the Dynabeads step was skipped and beads were washed straight away.

### TRAIL-R2 DISC IP

A total of 1.2 mg of anti-TRAIL-R2/DR5 antibody (Conatumumab, AMG655) (Amgen Inc.) was covalently attached to 6 mg of Dynabeads in a volume of 6 mL using the Dynabead® Antibody Coupling Kit (Life Technologies), according to the manufactory’s instructions. Typically, a P90 plate containing 10 mL of growth medium was inoculated with 30 µL of anti-DR5 beads when cells were approximately 60% confluent. The cells were then washed once in ice-cold PBS to slow down DISC dynamics and further procedures were carried out at 4 °C for the same reason. One milliliter of DISC IP buffer (20 mM Tris 7.4, 150 mM NaCl, 0.2% NP-40, 10% Glycerol, dH_2_O), supplemented with an EDTA free protease inhibitor cocktail, was added onto cells within the plate and allowed to shake for 60 min, then was transferred to a fresh tube. Anti-DR5 beads were isolated magnetically and washed 5 times with DISC IP buffer; the first flow-through was collected and termed the ‘Unbound Fraction’. Beads were then eluted using Loading buffer and analysed by Immunoblotting. AMG655 is an agonist antibody for TRAIL-R2DR5, therefore, activating the receptor, when it is immunoprecipitated after being added to live cells any complexes that associate with active DR5 (like the DISC) are also immunoprecipitated as well.

### Biotin pull down

For each sample, 25 µL of Dynabeads™ MyOne™ Streptavidin T1 (Invitrogen) were first washed in SDS-free RIPA buffer (50 mM Tris pH 7.4, 150 mM NaCl, 5 mM EDTA, 1% Triton X-100, dH_2_O) and then resuspended in SDS-Free RIPA + 2% BSA for blocking. 20 µg of the Biotin-tagged peptide (peptides&elephants) was then conjugated to the beads at room temperature of 30 min while rotating. The conjugated beads were then washed with SDS-Free RIPA buffer and incubated with 50 µg of cell lysate, collected in SDS-Free RIPA buffer supplemented with an EDTA free protease inhibitor cocktail, for 4 h at 4 °C while rotating. Beads were then washed with SDS-Free RIPA buffer and eluted in Loading buffer, followed by heating at 95 °C for 5 min. Samples were then analysed by immunoblotting.

### Site-directed mutagenesis

Site-directed mutagenesis was carried out using the KOD Xtreme™ Hot Start DNA Polymerase kit (Merck). Briefly, a PCR reaction (100 µL scale) incorporating 50 µL 2x buffer, 20 µL of 2 mM dNTPs, 6 µL each of 5 µM forward and reverse primers (containing the desired mutations), 16 µL of 10 ng/µL template DNA and 2 µL KOD polymerase were set up, according to the manufacturer’s instructions. Fifty microliters of the completed PCR reaction mixture was then digested with 2.5 µL Dpn1 and 5.8 µL CutSmart® Buffer (New England Biolabs) for 3 h at 37 °C to degrade methylated template DNA. CaCL_2_ competent DH5α bacteria were then transformed with the digested PCR mixture and plated onto agar with the appropriate selection marker. Subsequent colonies were then expanded, and clonal DNA was isolated using the QIAprep Spin Miniprep Kit (QIAGEN). Mutations in DNA were then verified by DNA sequencing (GATC BIOTECH).

### Caspase activity assay

Twenty-five microliters of Caspase-Glo®-3/7 or -8 reagent (Promega) was added to 5 µg (Caspase-3/7 activity) or 10 µg (Caspase-8 activity) of cell lysate, made up to 25 µL with PBS in a white-walled 96-well plate in duplicate. The plate was incubated in the dark at room temperature for 45 min and subsequently read at 1 s integrated reads in a luminescent plate reader (Biotek Synergy 4 plate reader). All conditions were performed in duplicate.

### High content fluorescent microscopy

This method was used to assess apoptotic cell death. Cells were seeded into a 96-well glass-bottom plate (Cellvis) and left to adhere overnight. After treatments, cells were incubated with 10x Annexin V Binding Buffer (made to 1×) (BD Pharmingen), 1:1000 FITC Annexin V (BD Pharmingen), 0.333 µg/mL Propidium Iodide (Sigma Aldrich) and 1.33 µg/mL Hoechst 33342 (Thermo Fisher Scientific) for 20 min at 37 °C. Images were then taken on the ArrayScan™ XTI HCA Reader, integrated with the CrEST™ X-Light™ Confocal Scan Head (Thermo Fisher Scientific) and the HCS Studio Cell Analysis Software V6.6.0 (Thermo Fisher Scientific) was used to analyse the images. Briefly, the software would recognise a cell as an object that was stained with Hoechst (blue), which stains all cells’ nuclei. Once the software had determined the cell as a valid object, it would detect whether the cell was also stained with Annexin V (AV:green), an indicator of early and late apoptosis, or Propidium Iodide (PI:red), an indicator of non-viable cells (Example images are in Supplementary Fig. 1a). The ArrayScan™ XTI HCA Reader would take multiple images from within a well until it had reached a target of 2,000 cells, or the maximum numbers of images had been taken for the given well. While images were being taken, the software would calculate the percentage of stained cells (either AV or PI or both), indicating total death, and this was plotted on a graph. All conditions were performed in triplicate.

### Sub-cellular fractionation

The ProteoExtract® Subcellular Proteome Extraction Kit (Calbiochem) was used to generate lysates from the cytosolic, nuclear and cytoskeletal/lipid raft fractions of the cell, according to the manufacturer’s instructions. The fractions were then subsequently analysed by immunoblotting.

### Protein half-life

Protein expression was analysed by Immunoblotting and quantified by densitometry, using ImageJ (NIH), normalising to the loading control. Treatment values were converted into ratios of its 0 h timepoint (given the value (1) and were plotted. GraphPad Prism 8 was then used to generate a decay curve for treatments, by using the one-phase exponential decay equation.

### Statistical analysis

Statistical significance was calculated from distinct technical replicates (*n* ≥ 3), either by student’s *T* test (two-tailed, two sample equal variance on unpaired data) or two-way ANOVA in GraphPad Prism 8. Graphs were plotted as means with error bars represented as SEM; statistical significance was denoted as follows: ****p < 0.0001, ***p < 0.001, **p < 0.01, *p < 0.05, ns = p > 0.05. Experimental phenotypes were confirmed in at least three independent experiments.

## Supplementary information

S1

S2

S3

S4

S5

Antibody Details

## Data Availability

The data supporting the findings of the study are available from the corresponding author on reasonable request.

## References

[1] Vaux DL, Korsmeyer SJ (1999). Cell death in development. Cell Cell Press.

[2] Taylor RC, Cullen SP, Martin SJ (2008). Apoptosis: controlled demolition at the cellular level. Nat Rev Mol Cell Biol.

[3] Humphreys L, Espona-Fiedler M, Longley DB. FLIP as a therapeutic target in cancer. FEBS J. 2018. http://www.ncbi.nlm.nih.gov/pubmed/29806737. 10.1111/febs.14523 29806737

[4] Riley JS, Malik A, Holohan C, Longley DB (2015). DED or alive: assembly and regulation of the death effector domain complexes. Cell Death Dis.

[5] Keller N, Mareš J, Zerbe O, Grütter MG (2009). Structural and biochemical studies on procaspase-8: new insights on initiator caspase activation. Structure.

[6] McLornan DP (2010). Prognostic significance of TRAIL signaling molecules in stage II and III colorectal cancer. Clin Cancer Res..

[7] McCarthy MM (2005). Evaluating the expression and prognostic value of TRAIL-R1 and TRAIL-R2 in breast cancer. Clin Cancer Res.

[8] Spierings DCJ (2003). Expression of TRAIL and TRAIL death receptors in stage III non-small cell lung cancer tumors. Clin Cancer Res.

[9] Leverkus M (2000). Regulation of tumor necrosis factor-related apoptosis-inducing ligand sensitivity in primary and transformed human keratinocytes. Cancer Res.

[10] Walczak H (1999). Tumoricidal activity of tumor necrosis factor-related apoptosis-inducing ligand in vivo. Nat Med.

[11] Tolcher AW (2007). Phase I pharmacokinetic and biologic correlative study of mapatumumab, a fully human monoclonal antibody with agonist activity to tumor necrosis factor-related apoptosis-inducing ligand receptor-1. J Clin Oncol.

[12] Plummer R (2007). Phase 1 and pharmacokinetic study of lexatumumab in patients with advanced cancers. Clin Cancer Res.

[13] Kumar S, Yoshida Y, Noda M (1993). Cloning of a cDNA which encodes a novel ubiquitin-like protein. Biochem Biophys Res Commun.

[14] Kumar S, Tomooka Y, Noda M (1992). Identification of a set of genes with developmentally down-regulated expression in the mouse brain. Biochem Biophys Res Commun.

[15] Hori T (1999). Covalent modification of all members of human cullin family proteins by NEDD8. Oncogene.

[16] Jones et al. A targeted proteomic analysis of the ubiquitin-like modifier nedd8 and associated proteins. J Proteome Res. 2018;7:1274–87. http://www.ncbi.nlm.nih.gov/pubmed/18247557. 10.1021/pr700749v PMC2676899 18247557

[17] Soucy TA (2009). An inhibitor of NEDD8-activating enzyme as a new approach to treat cancer. Nat.

[18] Petroski MD, Deshaies RJ. Function and regulation of cullin-RING ubiquitin ligases. Nat Rev Mol Cell Biol. 2005;6:9–20. http://www.ncbi.nlm.nih.gov/pubmed/15688063. 10.1038/nrm1547 15688063

[19] Wang Z, Liu P, Inuzuka H, Wei W (2014). Roles of F-box proteins in cancer. Nat Rev Cancer.

[20] Lafont E, Hartwig T, Walczak H (2017). Paving TRAIL’s path with ubiquitin. Trends Biochem Sci.

[21] Jin Z (2009). Cullin3-based polyubiquitination and p62-dependent aggregation of caspase-8 mediate extrinsic apoptosis signaling. Cell.

[22] Brownell JE (2010). Substrate-assisted inhibition of ubiquitin-like protein-activating enzymes: the NEDD8 E1 inhibitor MLN4924 forms a NEDD8-AMP mimetic in situ. Mol Cell.

[23] Wilson TR (2009). Combined inhibition of FLIP and XIAP induces Bax-independent apoptosis in type II colorectal cancer cells. Oncogene.

[24] Majkut J (2014). Differential affinity of FLIP and procaspase 8 for FADD’s DED binding surfaces regulates DISC assembly. Nat Commun.

[25] Gonzalvez F (2012). TRAF2 sets a threshold for extrinsic apoptosis by tagging caspase-8 with a Ubiquitin shutoff timer. Mol Cell.

[26] Schuler S (2011). SKP2 confers resistance of pancreatic cancer cells towards TRAIL-induced apoptosis. Int J Oncol.

[27] Hughes MA (2016). Co-operative and hierarchical binding of c-FLIP and caspase-8: a unified model defines how c-FLIP isoforms differentially control cell fate. Mol Cell.

[28] Henry CM, Martin SJ (2017). Caspase-8 acts in a non-enzymatic role as a scaffold for assembly of a pro-inflammatory “FADDosome” complex upon TRAIL stimulation. Mol Cell.

[29] Yu JW, Jeffrey PD, Shi Y (2009). Mechanism of procaspase-8 activation by c-FLIPL. Proc Natl Acad Sci USA.

[30] Humphreys LM, et al. A revised model of TRAIL ‐R2 DISC assembly explains how FLIP (L) can inhibit or promote apoptosis. EMBO Rep. 2020;21:e49254. 10.15252/embr.201949254. 10.15252/embr.201949254 PMC7054686 32009295

[31] Carson R (2015). HDAC inhibition overcomes acute resistance to MEK inhibition in BRAF-mutant colorectal cancer by downregulation of c-FLIPL. Clin Cancer Res.

[32] Crawford N (2013). SAHA overcomes FLIP-mediated inhibition of SMAC mimetic-induced apoptosis in mesothelioma. Cell Death Dis.

[33] Paul I (2012). Acquired differential regulation of caspase-8 in cisplatin-resistant non-small-cell lung cancer. Cell Death Dis.

[34] McLaughlin KA (2016). FLIP: a targetable mediator of resistance to radiation in non-small cell lung cancer. Mol Cancer Ther.

[35] Poukkula M (2005). Rapid turnover of c-FLIPshort is determined by its unique C-terminal tail. J Biol Chem.

[36] Mohr A (2018). Caspase-10: a molecular switch from cell-autonomous apoptosis to communal cell death in response to chemotherapeutic drug treatment. Cell Death Differ.

[37] Chang L (2006). The E3 ubiquitin ligase itch couples JNK activation to TNFα-induced cell death by inducing c-FLIPL turnover. Cell..

[38] Santini S (2014). ATM kinase activity modulates ITCH E3-ubiquitin ligase activity. Oncogene..

[39] Bellail AC, Olson JJ, Yang X, Chen ZJ, Hao C (2012). A20 ubiquitin ligase-mediated polyubiquitination of RIP1 inhibits caspase-8 cleavage and TRAIL-induced apoptosis in glioblastoma. Cancer Discov.

[40] Xu L (2017). DR5-Cbl-b/c-Cbl-TRAF2 complex inhibits TRAIL-induced apoptosis by promoting TRAF2-mediated polyubiquitination of caspase-8 in gastric cancer cells. Mol Oncol..

[41] Tschopp J, Kataoka T (2004). N-terminal fragment of c-FLIP(L) processed by caspase 8 specifically Interacts with TRAF2 and induces activation of the NF-kB signaling pathway. Mol Cell Biol.

