# Supplementary figures and images for: The SCFSkp2 ubiquitin ligase complex modulates TRAIL-R2-induced apoptosis by regulating FLIP(L)
Source: Cell Death Differ. 2020 Apr 20;27(9):2726–41. doi: 10.1038/s41418-020-0539-7 (PMC7429845; doi:10.1038/s41418-020-0539-7)

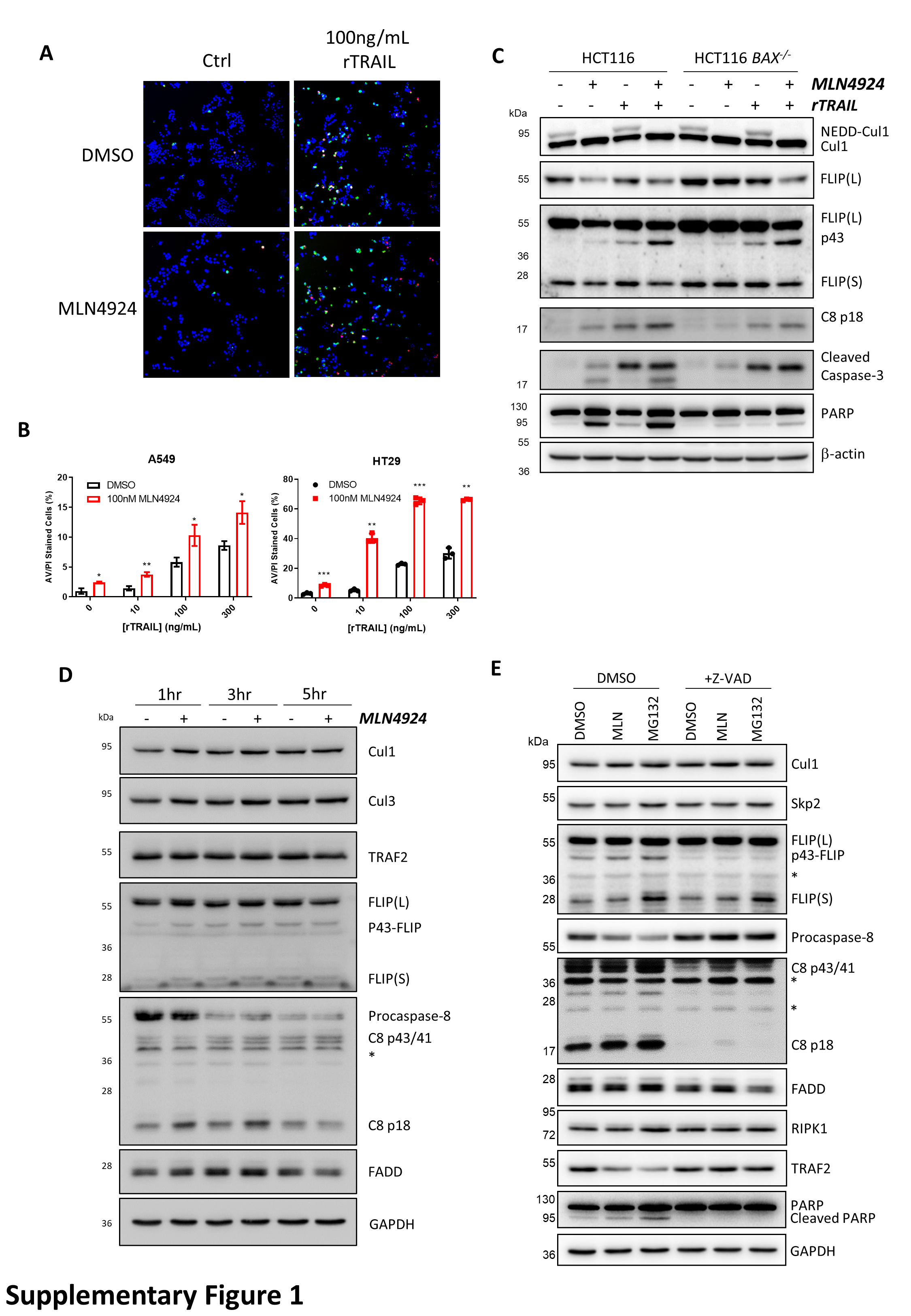

Supplement: Supplementary file 1 — S1 [file 41418_2020_539_MOESM1_ESM.tif]

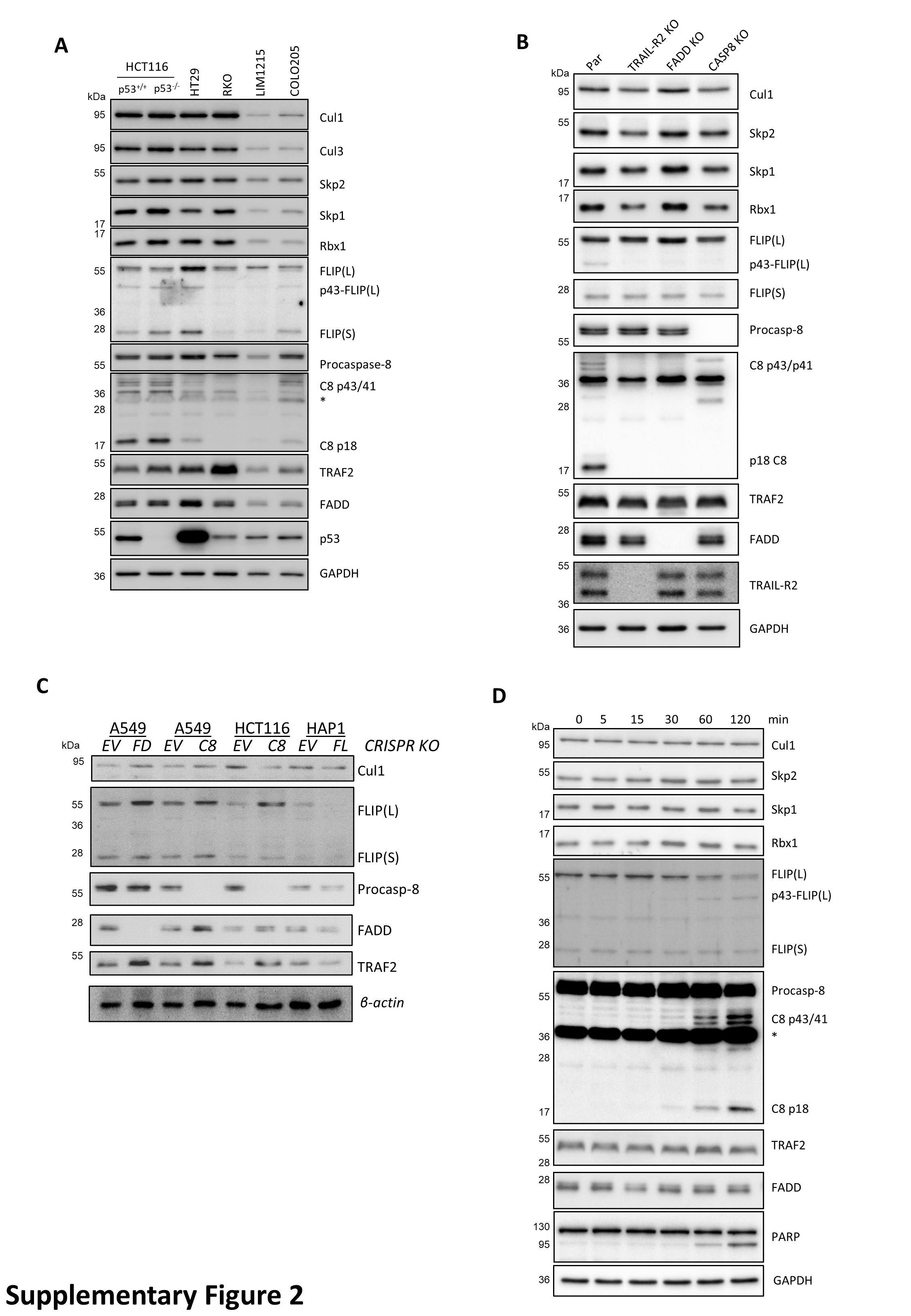

Supplement: Supplementary file 2 — S2 [file 41418_2020_539_MOESM2_ESM.tif]

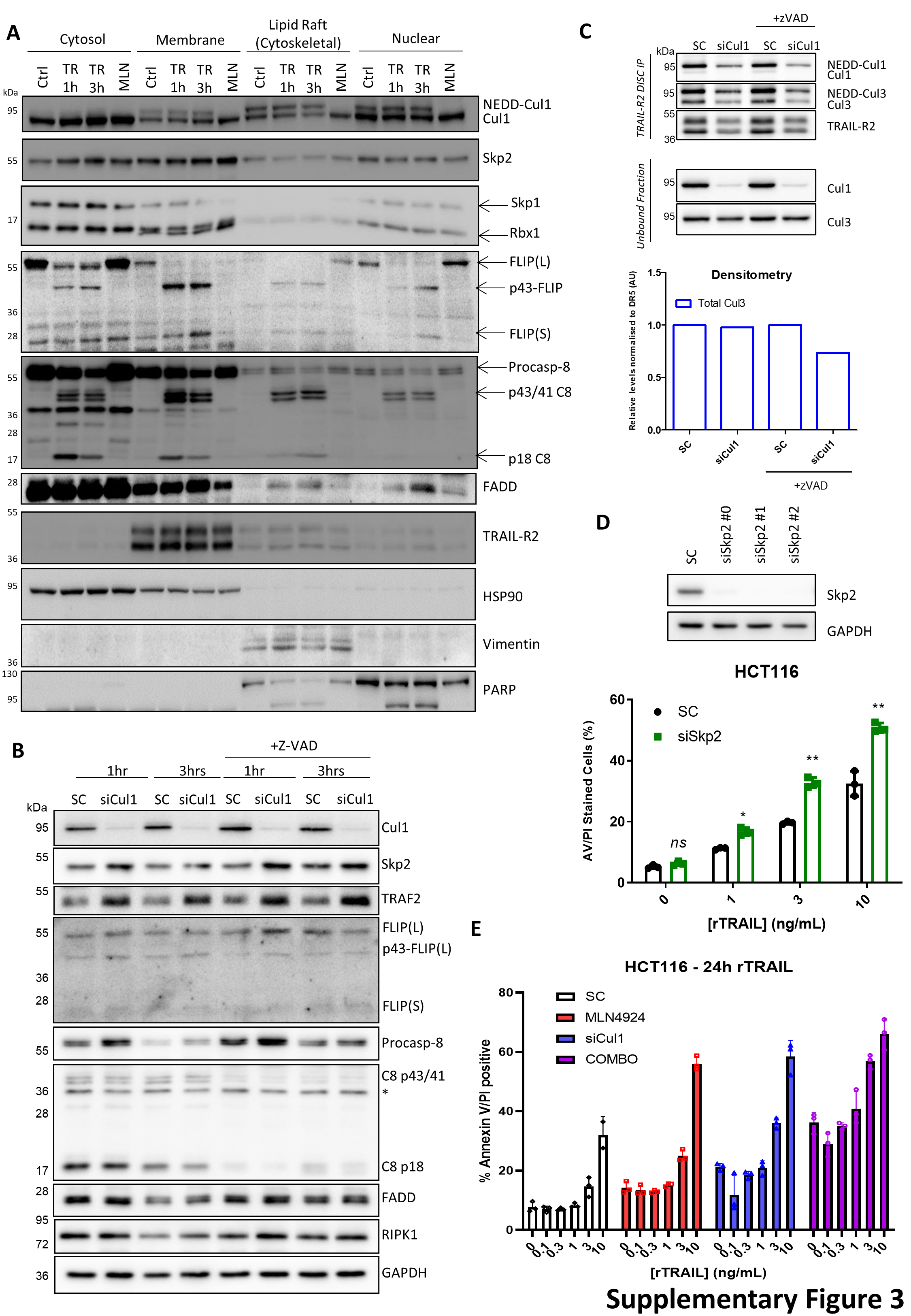

Supplement: Supplementary file 3 — S3 [file 41418_2020_539_MOESM3_ESM.tif]

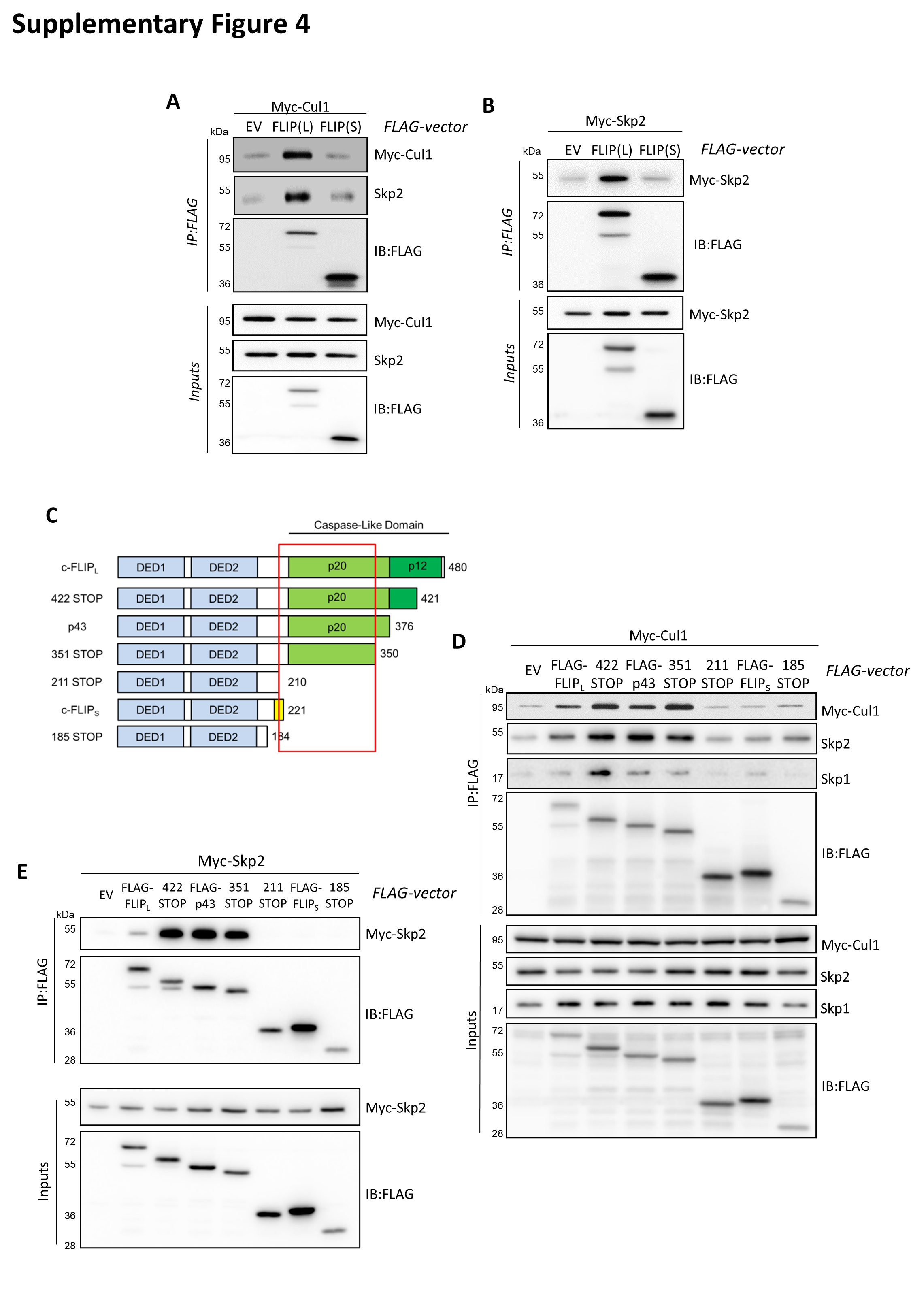

Supplement: Supplementary file 4 — S4 [file 41418_2020_539_MOESM4_ESM.tif]

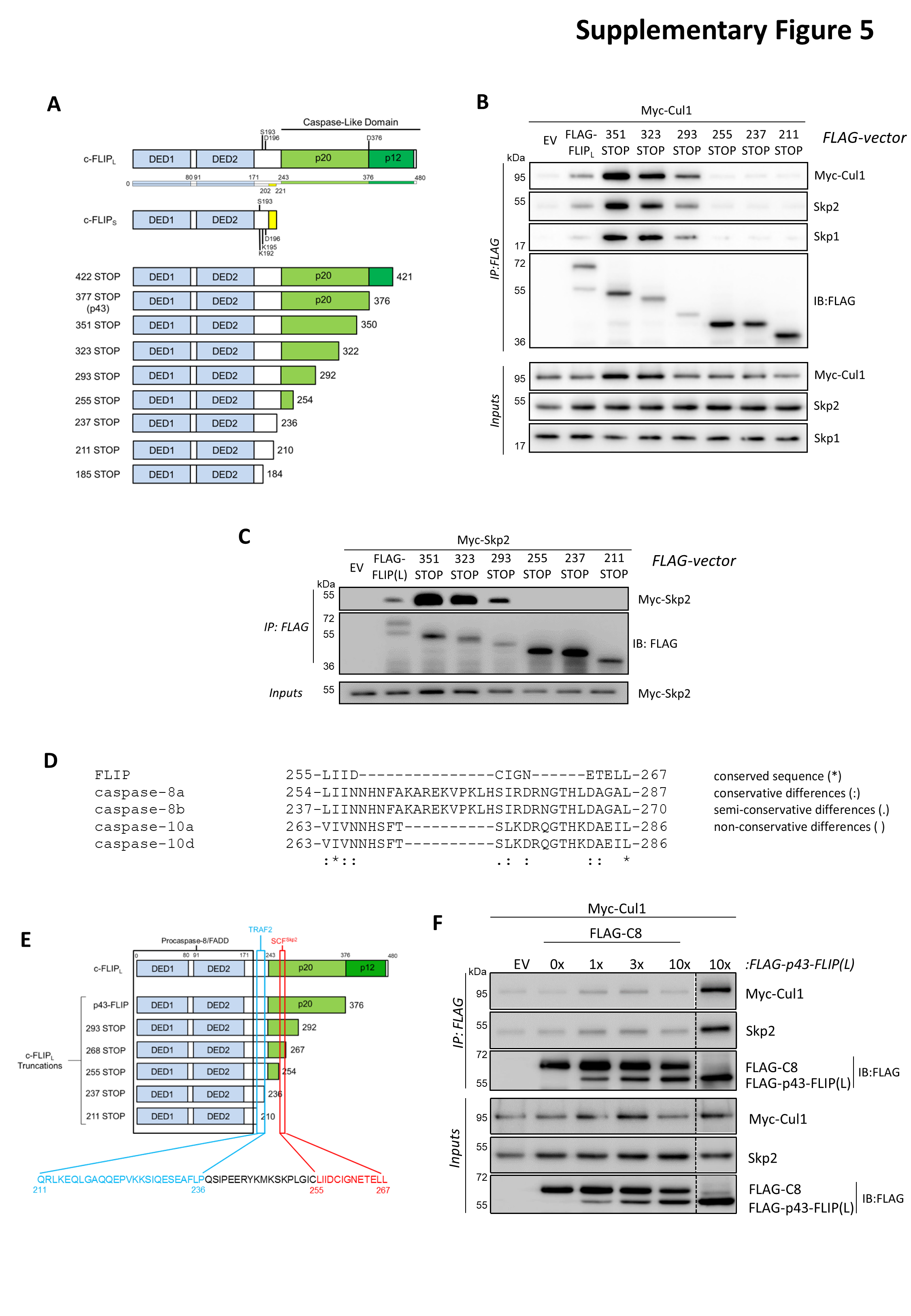

Supplement: Supplementary file 5 — S5 [file 41418_2020_539_MOESM5_ESM.tif]
