# Supplementary material for: The SCFSkp2 ubiquitin ligase complex modulates TRAIL-R2-induced apoptosis by regulating FLIP(L)
Source: Cell Death Differ. 2020 Apr 20;27(9):2726–41. doi: 10.1038/s41418-020-0539-7 (PMC7429845; doi:10.1038/s41418-020-0539-7)
Supplement: Supplementary file 6 — Antibody Details [file 41418_2020_539_MOESM6_ESM.docx]

**Supplementary Table**

**Antibodies.**

| **REAGENTS** | **SOURCE** | **IDENTIFIER** |
| --- | --- | --- |
| **Imunnoblot Antibodies** | | |
| β-actin (AC-74) | Sigma | A5316 |
| AMG655 | Amgen Inc. | NA |
| Anti-Mouse HRP | Cell Signaling Technology | 7076 |
| Anti-Rabbit HRP | Cell Signaling Technology | 7074 |
| Bax | Cell Signaling Technology | 2774 |
| Caspase-3 | Cell Signaling Technology | 9662 |
| Caspase-8 (12F5) | Enzo | ALX-804-242-C100 |
| Caspase-8 (E6) | abcam | ab32125 |
| Cullin1 (19) | BD Biosciences | 612040 |
| Cullin3 (3) | BD Biosciences | 611848 |
| DR5 (D4E9) XP | Cell Signaling Technology | 8074 |
| Dynabeads™ M-280 Anti-Rabbit | Invitrogen | 11204D |
| Dynabeads™ M-280 Anti-Mouse | Invitrogen | 11201D |
| FADD (H-181) | Santa Cruz Biotechnology | sc-5559 |
| FADD (A66-2) | BD Biosciences | 556402 |
| FLAG (M2) HRP | Sigma-Aldrich | A8592 |
| FLAG (M2) Magnetic Beads | Sigma-Aldrich | M8823 |
| FLIP(H-202) | Santa Cruz Biotechnology | Sc-8347 |
| FLIP (NF6) | AdipoGen | AG-20B-0056-C100 |
| GAPDH (6C5) | abcam | ab8245 |
| HSP90 (H-114) | Santa Cruz Biotechnology | sc-7947 |
| Myc Tag (71D10) | Cell Signaling Technology | 2278 |
| Myc-Tag (9B11) | Cell Signaling Technology | 2276 |
| PARP | Cell Signaling Technology | 9542 |
| p21 (C-19) | Santa Cruz Biotechnology | sc-397 |
| p53 (DO-1) | Santa Cruz Biotechnology | sc-126 |
| Rbx1 (D3J5I) | Cell Signaling Technology | 11922 |
| Rabbit IgG Control | Santa Cruz Biotechnology | sc-2027 |
| RIP/RIPK1 (38) | BD Biosciences | 610458 |
| Skp1 (D3J4N) | Cell Signaling Technology | 12248 |
| Skp2 (D3G5) | Cell Signaling Technology | 2652 |
| Skp2 (SKP2-8D9) | Invitrogen | 32-3300 |
| TRAF2 (C192) | Cell Signaling Technology | 4724 |
| TRAF2 (C90-481) | BD Biosciences | 558890 |
| Ubiquitin (P4D1) | Santa Cruz Biotechnology | sc-8017 |
| Ubiquitin | Cell Signaling Technology | 3933 |
| Vimentin (V9) | Santa Cruz Biotechnology | sc-6260 |
|  |  |  |
